# Human Granulocyte Macrophage Colony-Stimulating Factor Enhances Antibiotic Susceptibility of *Pseudomonas aeruginosa* Persister Cells

**DOI:** 10.1038/srep17315

**Published:** 2015-11-30

**Authors:** Geetika S. Choudhary, Xiangyu Yao, Jing Wang, Bo Peng, Rebecca A. Bader, Dacheng Ren

**Affiliations:** 1 Department of Biomedical and Chemical Engineering, Syracuse University, Syracuse, NY 13244, USA; 2 Syracuse Biomaterials Institute, Syracuse University, Syracuse, NY 13244, USA; 3 Department of Civil and Environmental Engineering, Syracuse University, Syracuse, NY 13244, USA; 4 Department of Biology, Syracuse University, Syracuse, NY 13244, USA

## Abstract

Bacterial persister cells are highly tolerant to antibiotics and cause chronic infections. However, little is known about the interaction between host immune systems with this subpopulation of metabolically inactive cells, and direct effects of host immune factors (in the absence of immune cells) on persister cells have not been studied. Here we report that human granulocyte macrophage-colony stimulating factor (GM-CSF) can sensitize the persister cells of *Pseudomonas aeruginosa* PAO1 and PDO300 to multiple antibiotics including ciprofloxacin, tobramycin, tetracycline, and gentamicin. GM-CSF also sensitized the biofilm cells of *P. aeruginosa* PAO1 and PDO300 to tobramycin in the presence of biofilm matrix degrading enzymes. The DNA microarray and qPCR results indicated that GM-CSF induced the genes for flagellar motility and pyocin production in the persister cells, but not the normal cells of *P. aeruginosa* PAO1. Consistently, the supernatants from GM-CSF treated *P. aeruginosa* PAO1 persister cell suspensions were found cidal to the pyocin sensitive strain *P. aeruginosa* PAK. Collectively, these findings suggest that host immune factors and bacterial persisters may directly interact, leading to enhanced susceptibility of persister cells to antibiotics.

First defined in 1940 s^1^, persister cells are a small fraction (normally less than 1% of the total population) of metabolically inactive bacterial cells that are phenotypic variants and are highly tolerant to antibiotics and other environmental stresses. When an antibiotic therapy is stopped, the surviving persisters can relapse to metabolically active normal cells causing chronic infections with recurring symptoms^2^. SOS responses to DNA damage, amino acid starvation, oxidative stress, change in nutritional source, and other stresses are all known to induce persister formation^3^. At the molecular level, toxin-antitoxin (TA) modules have been shown to play a pivotal role in persister formation^4^. Each TA module encodes a “toxin” that disrupts specific cellular process(es) and a corresponding “antitoxin” that neutralizes the toxin^4^,^5^. Thus, unbalanced TA production and accumulation of free toxins can lead to persister formation^6^. In general, the population of persister cells is higher in stationary phase cultures and in biofilms, which are complex communities of cells attached to surfaces with the protection of an extracellular matrix secreted by the attached cells^7^,^8^. Biofilms are involved in 80% of bacterial infections in humans and biofilm cells are up to 1000 times more tolerant to antibiotics than normal planktonic cells^7^,^9^.

During bacterial infection, the host innate immunity acts as the first line of defense to block the entry of pathogens and kill the microbes that successfully penetrate the epithelial barrier^10^. As an innate immune response, macrophages and dendritic cells secrete cytokines, which are signaling proteins acting as mediators to attract more immune cells, such as phagocytes^11^. Pathogen-associated molecular patterns (PAMPs) found on microorganisms are identified by pattern recognition receptors (PRRs)^12^ present on the surface and in the cytoplasm of innate immune cells such as macrophages, dendritic cells, and natural killer cells^13^. The recognition of pathogens is followed by their elimination by phagocytosis involving uptake of pathogens in phagosomes and macropinocytosis involving uptake of macromolecules and extracellular fluid^11^,^14^,^15^.

Macrophages secrete various cytokines such as IL-1, IL-6, IL-8, IL-10, IL-11, IL-12, IL-15, TNF-α, IFN- α&β, M-CSF, GM-CSF, and G-CSF^16^. Among them, GM-CSF (granulocyte macrophage colony-stimulating factor) is a cytokine secreted by macrophages, T-cells, mast cells, NK cells, endothelial cells, and fibroblasts; and is considered as a major regulator governing the maturation of granulocytes and macrophages^17^,^18^. The level of human GM-CSF in the circulation under normal conditions is around 0.17 ± 0.03 pM^19^. However, it increases in response to infection to help recruit monocytes/macrophages^20^. Gonzalez-Juarrero *et al*.^21^ showed that GM-CSF knockout (KO) mice succumb to pulmonary infection by *Mycobacterium tuberculosis* faster than the mice with GM-CSF expression in the lungs (GM^+^). Recently, the use of immunotherapeutic agents are being explored for the treatment of drug resistant Tuberculosis (TB) caused by *M. tuberculosis*^22^. Nambiar *et al*.^23^ demonstrated that delivery of GM-CSF to the lungs of immunodeficient mice through GM-CSF secreting *Mycobacterium bovis* BCG vaccine strain (BCG:GM-CSF) led to increase in pulmonary dendritic cell numbers and 10-fold more efficient clearance of *M. tuberculosis* H37Rv. These studies demonstrated that GM-CSF is actively involved in host immune response to the invasion of pathogens.

Compared to the well documented studies on cytokine production and the functions of cytokines in stimulating host immune cells, little is known about how bacteria respond to these host signaling molecules. Kanangat *et al*.^24^ studied intracellular growth of *Staphylococcus aureus*, *P. aeruginosa*, and *Acinetobacter sp*. (6 × 10^6^ CFU of each strain) added to monocytic cells primed with low doses (0, 10, 100, and 250 pg) and high doses (1 and 10 ng) of IL-1 β, IL-6, and TNF-α. It was found that at low cytokine doses (10 to 250 pg), the intracellular bacterial growth of all strains decreased; however, as the dose increased to 10 ng, the trend reversed^24^. It was speculated that above a threshold concentration of cellular activation, the conditions become favorable for the survival and replication of ingested bacteria^24^.

To our best knowledge, the interaction between antibiotic tolerant persister cells and host cytokines has not been explored. Motivated by this knowledge gap, we conducted this study to investigate the effects of cytokine GM-CSF on *P. aeruginosa* persister cells. We selected *P. aeruginosa* as the model bacterium because it is a widely used organism for research on persister cells and biofilms^25^,^26^. Effects of GM-CSF on *P. aeruginosa* PAO1 and the mucoid strain PDO300 were compared with GM-CSF introduced either alone or with an antibiotic together to test synergy. PDO300 is a *mucA22* mutant (due to a single base pair deletion) of *P. aeruginosa* PAO1, which overproduces the exopolysaccharide alginate^27^. Alginate overproduction also leads to mucoidity, which is commonly seen in late stage cystic fibrosis patients with multidrug tolerant infections^28^. The non-pathogenic laboratory strain *E. coli* K12 and pathogenic strain *E. coli* ATCC 53505 were also included in this study to understand if GM-CSF has different activities on these strains.

## Results

### Persister isolation

Unlike antibiotic resistant mutants that are based on acquired genetic elements and can grow in the presence of antibiotics, persistence is a reversible physiological state^29^,^30^. This characteristic enables the subpopulation of persisters to survive the killing by antibiotics, resuming growth and normal antibiotic susceptibility when antibiotics are removed^29^,^30^. Thus, the presence of persister cells is evidenced by biphasic killing by antibiotics, which has a rapid killing of normal cells and a plateau with surviving persisters irresponsive to very high concentrations of antibiotics even with prolonged treatment. This was observed for *P. aeruginosa* PAO1, PDO300, *E. coli* K12, and *E. coli* ATCC 53505 cells isolated from both exponential and stationary phase cultures (Supplementary Fig. S1), confirming the presence of persister cells in our experimental conditions. Based on these results, we selected 3.5 h treatment time and high concentrations of antibiotics (200 μg/mL ciprofloxacin for *P. aeruginosa* PAO1 and PDO300; 100 μg/mL ampicillin for *E. coli* K12; 20 μg/mL ofloxacin for *E. coli* ATCC 53505) to isolate persister cells. These conditions are consistent with previous reports in literature^8^,^31^ and the selected concentration for isolating *P. aeruginosa* persister cells is at least 10-fold higher than the MIC of these strains (Supplementary Table S1).

### GM-CSF sensitized the planktonic persister cells of *P. aeruginosa* PAO1 to antibiotics

**Figure 1:**
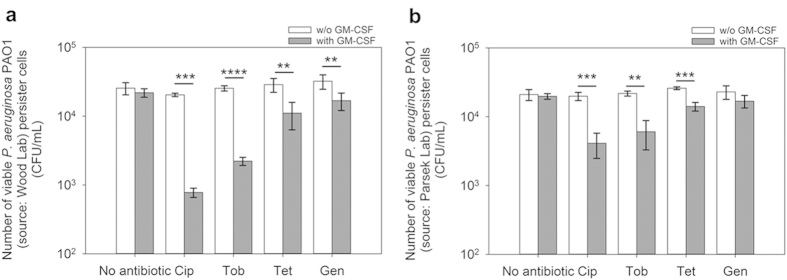
GM-CSF sensitized the persister cells of *P. aeruginosa* PAO1 to antibiotics. The wild-type PAO1 obtained from two different sources were tested including one (**a**) from Prof. Thomas K. Wood at Pennsylvania State University and another (**b**) from Prof. Matthew Parsek at University of Washington. The persister cells were isolated from exponential phase cultures by killing the normal cells with 200 μg/mL ciprofloxacin for 3.5 h, and then treated with 0.17 pM GM-CSF alone for 1 h, followed by additional treatment with GM-CSF plus an antibiotic as indicated for 3.5 h (all tested at 200 μg/mL). The samples without GM-CSF or antibiotic were used as controls. The amount of BSA (0.1%) was adjusted to be the same for all samples. Following the treatment, the viability of persister cells was determined by counting CFU. Cip: ciprofloxacin. Tob: tobramycin. Tet: tetracycline. Gen: gentamicin. The samples were tested in triplicate (n = 3). Error bars represent SD; **p < 0.01, ***p < 0.001, ****p < 0.0001, one-way ANOVA followed by Tukey test.

We started this study using the *P. aerugionsa* PAO1 strain^32^ obtained from Prof. Thomas K. Wood at Pennsylvania State University. Treatment of this strain with 0.17 pM GM-CSF alone did not affect the viability of persister cells isolated (by treatment with 200 μg/mL ciprofloxacin for 3.5 h) from the exponential phase cultures [p = 0.36; One-way ANOVA followed by Tukey test used throughout this study] (Fig. 1a). However, the treatment with GM-CSF significantly sensitized the persister cells to antibiotics. For example, treatment with 0.17 pM recombinant human GM-CSF (henceforth GM-CSF) sensitized 96.2 ± 5.9% (26.3-fold reduction of viability; p = 0.0002), 91.3 ± 1.2% (p < 0.0001), 61.4 ± 16.6% (p = 0.0119), and 47.6 ± 14.9% (p = 0.0030) of the persister cells to 200 μg/mL of ciprofloxacin, tobramycin, tetracycline, and gentamicin respectively, compared to treatments with antibiotic alone, which were found ineffective in killing persister cells (p > 0.05 for all antibiotics tested) (Fig. 1a). To test if GM-CSF is also effective against the mucoid strains of *P. aeruginosa*, we tested another wild-type strain of *P. aeruginosa* PAO1^33^ obtained from Prof. Matthew Parsek at the University of Washington, so that the isogenic mucoid strain *P. aeruginosa* PDO300^33^ can be compared. Similar results were obtained for the PAO1 strain from Parsek lab; e.g., treatment with 0.17 pM GM-CSF alone did not affect the viability of persister cells isolated from exponential phase cultures (p = 0.37) (Fig. 1b), but sensitized the persister cells to the above antibiotics except for gentamycin. For example, treatment with 0.17 pM GM-CSF for 1 h sensitized 79.3 ± 8.3% (p = 0.0002), 72.2 ± 12.7% (p = 0.0012), and 45.7 ± 7.8% (p = 0.001) of persister cells to 200 μg/mL of ciprofloxacin, tobramycin, and tetracycline, respectively, compared to treatments with antibiotic alone (Fig. 1b). Treatment with any of these antibiotics alone did not cause significant killing of persister cells (p > 0.05 for all). Since the results are generally consistent with the other PAO1 strain and an isogenic mucoid strain is available, the PAO1 strain from Parsek lab (henceforth PAO1) was used for the rest of this study. Furthermore, because persister population is larger in stationary phase, we also tested if GM-CSF is effective against persister cells isolated from stationary phase cultures. The concentrations of antibiotics were also reduced to better understand the potential of GM-CSF. As shown in Table 1, GM-CSF itself did not significantly affect the viability of persisters in the absence of an antibiotic (0.17, 1.7, and 17 pM GM-CSF tested; p > 0.3 for all conditions), and synergistic effects were observed between GM-CSF and antibiotics in killing PAO1 persister cells isolated from stationary phase cultures. Specifically, treatment with 0.17 pM GM-CSF sensitized 61.5 ± 14.5% (p = 0.0003) and 77.1 ± 2.0% (p = 0.0048) of persister cells to 5 μg/mL ciprofloxacin and tobramycin respectively, compared to treatments with antibiotic alone (Table 1). At a higher concentration of 17 pM, GM-CSF sensitized 74.0 ± 2.9% (p = 0.0005) and 86.5 ± 1.7% (p = 0.0002) of PAO1 persister cells to 5 μg/mL ciprofloxacin and tobramycin, respectively, compared to antibiotic treatments alone (Table 1). Thus, GM-CSF appeared to be effective against persister cells in both exponential and stationary phase cultures of *P. aeruginosa* PAO1 and exhibited synergy in persister killing with low concentrations of ciprofloxacin and tobramycin.

**Figure 2:**
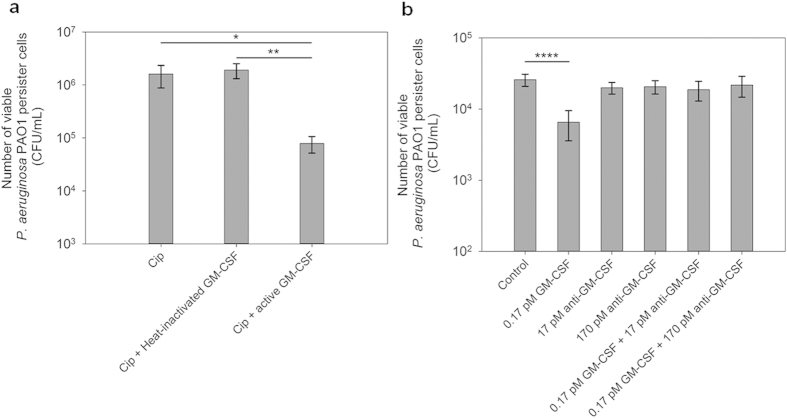
Effect of GM-CSF on *P. aeruginosa* PAO1 persister cells was abolished by heat inactivation and anti-GM-CSF. (**a**) The persister cells isolated from stationary phase cultures underwent treatment with active 0.17 pM GM-CSF or heat-inactivated 0.17 pM GM-CSF in the presence of 5 μg/mL ciprofloxacin for 3.5 h. (**b**) The persister cells were isolated from exponential phase cultures. All samples underwent the same incubation time. The figure shows the viability of persister cells treated with GM-CSF alone, anti-GM-CSF alone, or GM-CSF neutralized by anti-GM-CSF (2 h incubation) followed by addition of 5 μg/mL ciprofloxacin and incubation for 3.5 h. The amount of BSA (0.1%) was adjusted to be the same for all samples. Following the treatment, the viability of persister cells was determined by counting CFU. The samples were tested in triplicate (n = 3). Error bars represent SD; * p < 0.05, ** p < 0.01,**** p < 0.0001, one-way ANOVA followed by Tukey test.

To confirm if the observed effects were caused by GM-CSF rather than any contaminant, we inactivated the GM-CSF stock by heating at 75 °C overnight. This treatment abolished the effects of GM-CSF on *P. aeruginosa* PAO1 persister cells during treatment with ciprofloxacin (Fig. 2a). This finding is consistent with the heat sensitive nature of proteins. To further confirm that the observed effects were caused by GM-CSF specifically, we also tested the effects in the presence of anti-GM-CSF antibody. As shown in Fig. 2b, pretreatment of GM-CSF with anti-GM-CSF (2 h incubation) abolished the effects of GM-CSF. Thus, the observed effects on persister cells were indeed caused by GM-CSF.

### GM-CSF is effective against the mucoid strain PDO300 in the presence of alginate lyase

**Figure 3:**
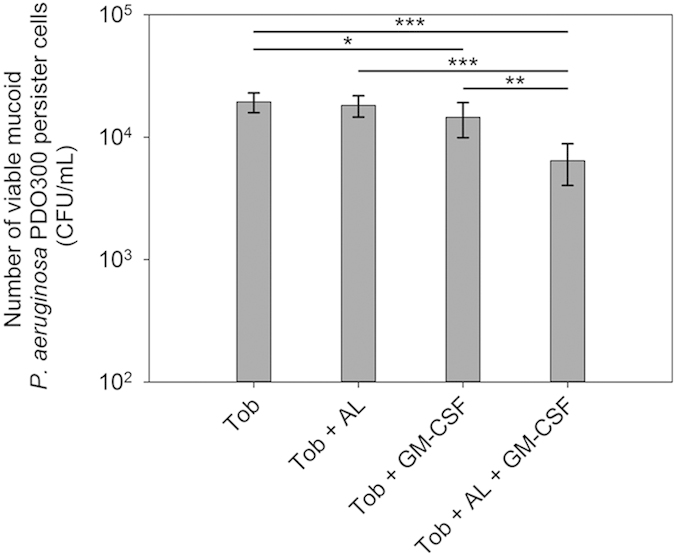
Alginate lyase is required for the activity of GM-CSF against persister cells of the mucoid strain *P. aeruginosa* PDO300. The persister cells were isolated from exponential phase cultures and GM-CSF was tested at 0.17 pM. The viability of persister cells treated with tobramycin (200 μg/mL) alone, tobramycin along with alginate lyase (50 μg/mL), tobramycin along with GM-CSF, or tobramycin along with alginate lyase and GM-CSF is shown. The amount of BSA (0.1%) was adjusted to be the same for all samples. Following the treatment, the viability of persister cells was determined by counting CFU. Tob: tobramycin. AL: alginate lyase. The samples were tested in triplicate (n = 3). Error bars represent SD; *p < 0.05, **p < 0.01, ***p < 0.001, one-way ANOVA followed by Tukey test.

To understand if GM-CSF also affects the mucoid strains of *P. aeruginosa*, the persister cells of *P. aerugionsa* PDO300 (henceforth PDO300) isolated from exponential phase cultures were tested following the same protocol used for PAO1 cells. Similar to the results of the wild-type PAO1, treatment with 0.17 pM GM-CSF did not change the viability of PDO300 persister cells (p = 0.77), but sensitized 40.5 ± 18.6% (p = 0.04) of persister cells to 200 μg/mL tetracycline, compared to treatment with tetracycline alone. The decrease in activities of GM-CSF against PDO300 persister cells compared to PAO1 is probably due to the presence of its alginate layer since when 50 μg/mL alginate lyase was added, the reduction in the viability by 200 μg/mL tobramycin and 0.17 pM GM-CSF increased significantly (Fig. 3). Specifically, co-treatment with tobramycin, GM-CSF, and alginate lyase led to killing of persister cells that was 66.9 ± 12.4% (p = 0.0002), 55.7 ± 16.5% (p = 00031), and 64.6 ± 13.2% (p = 0.0003) more than killing by tobramycin alone, tobramycin + GM-CSF, and tobramycin + alginate lyase, respectively (Fig. 3). Prior to the experiment, we had verified that 10, 50, 100, and 200 μg/mL of alginate lyase itself had insignificant (p > 0.05) effect on persister cells of *P. aeruginosa* PDO300. Overall, these results indicate that GM-CSF can also sensitize persister cells of the mucoid strain *P. aeruginosa* PDO300 to tobramycin if alginate lyase is present. We believe this is because alginate lyase can help GM-CSF penetrate alginate, as shown in our recent Western results^34^.

### GM-CSF enhanced the killing of biofilm cells in the presence of biofilm matrix degrading enzymes

**Figure 4:**
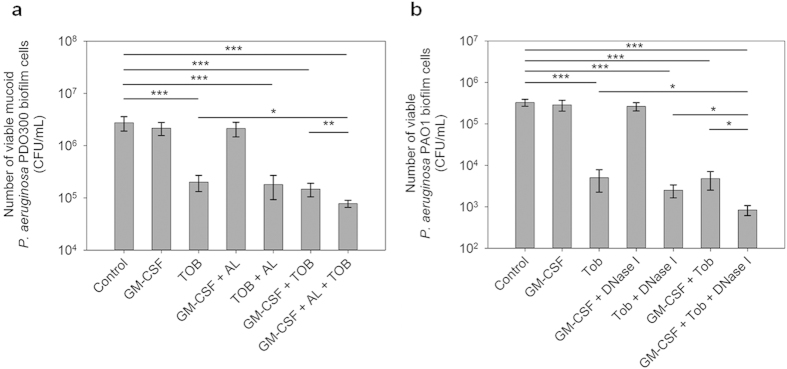
GM-CSF enhanced the killing of *P. aeruginosa* PDO300 and PAO1 biofilm cells by tobramycin in the presence of alginate lyase and DNase I respectively. (**a**) *P. aeruginosa* PDO300 cells in 24 h biofilms were treated with (**i**) 0.17 pM GM-CSF alone, (**ii**) 200 μg/mL tobramycin alone, (**iii**) 0.17 pM GM-CSF and 50 μg/mL alginate lyase, (**iv**) 200 μg/mL tobramycin and 50 μg/mL alginate lyase, (**v**) 0.17 pM GM-CSF and 200 μg/mL tobramycin, and (**vi**) 0.17 pM GM-CSF, 200 μg/mL tobramycin, and 50 μg/mL alginate lyase, for 3.5 h. Tob: tobramycin. AL: alginate lyase. (**b**) *P. aeruginosa* PAO1 cells in early biofilms (4 h after inoculation) were treated with (**i**) 0.17 pM GM-CSF alone, (**ii**) 20 μg/mL tobramycin alone, (**iii**) 0.17 pM GM-CSF and 5 units/mL DNase I, (**iv**) 20 μg/mL tobramycin and 5 units/mL DNase I, (**v**) 0.17 pM GM-CSF and 20 μg/mL tobramycin, and (**vi**) 0.17 pM GM-CSF, 20 μg/mL tobramycin, and 5 units/mL DNase I for 3.5 h. For both PAO1 and PDO300 biofilm cells, the amount of BSA (0.1%) was adjusted to be the same for all samples. Following the treatment, the viability of biofilm cells was determined by counting CFU. The samples were tested in triplicate (n = 3). Error bars represent SD; *p < 0.05, **p < 0.01, ***p < 0.001, one-way ANOVA followed by Tukey test.

To understand if GM-CSF is also effective against *P. aeruginosa* biofilm cells, the 24 h biofilm cells of PAO1 and PDO300 were treated with GM-CSF in the presence and absence of antibiotics. Treatment with 0.17 pM GM-CSF alone did not change the viability of biofilm cells of either strain. Since alginate can hinder the penetration of GM-CSF^34^. Alginate lyase was added at 50 μg/mL in addition to 200 μg/mL tobramycin and 0.17 pM GM-CSF to test the effects of GM-CSF on biofilm cells. Neither GM-CSF nor alginate lyase alone killed biofilm cells of *P. aeruginosa* PAO1 and PDO300 significantly in the absence of antibiotic. However, co-treatment with 50 μg/mL alginate lyase, 0.17 pM GM-CSF, and 200 μg/mL tobramycin reduced the viability of PDO300 biofilm cells by 97.2 ± 0.4% (35.4-fold reduction of viability; p = 0.0002) compared to the control biofilms without any treatment (Fig. 4a). This is 61.3 ± 6.0% (p = 0.03) more killing than that achieved by treatment with tobramycin alone, and 57.1 ± 6.6% more than treatment with tobramycin and alginate lyase together (Fig. 4a). Thus, GM-CSF is effective against PDO300 biofilm cells if the biofilm matrix is degraded.

In comparison, addition of alginate lyase did not exhibit synergistic effects between GM-CSF and antibiotics in killing *P. aeruginosa* PAO1 biofilm cells. For example, addition of 0.17 pM GM-CSF and 50 μg/mL alginate lyase did not further reduce the viability of biofilm cells compared to treatment with 200 μg/mL tobramycin alone (p = 0.82). This is consistent with the report by Wozniak *et al*.^35^ that alginate is not a major component of the extracellular polysaccharide matrix of the wild-type PAO1. Since early PAO1 biofilms are known to contain a large amount of extracellular DNA, we tested if addition of DNase I could increase the activity of GM-CSF in killing early stage (4 h) PAO1 biofilm cells. It was found that DNase I alone at 1, 2, 5, or 10 units/mL had no significant effects (p > 0.05) on the viability of biofilm cells. However, addition of DNase I along with GM-CSF and tobramycin was found to kill *P. aeruginosa* PAO1 biofilm cells significantly. For example, when 5 units/mL DNase I and 0.17 pM GM-CSF were added along with 20 μg/mL tobramycin (lower antibiotic concentration was used since this was tested on early stage biofilms), the viability of biofilm cells was reduced by 99.7 ± 0.1% (near 3 log reduction of viability; p = 0.0008) compared to the untreated control (Fig. 4b). This is 83.3 ± 4.5% (p = 0.05) more than treatment with tobramycin alone, and 66.4 ± 9.1% (p = 0.0203) more than treatment with tobramycin and DNase I together (Fig. 4b). Collectively, the above findings indicate that GM-CSF is also effective against biofilm cells if the biofilm matrix is removed.

### GM-CSF is not effective against normal planktonic cells of *P. aeruginosa* PAO1 and PDO300

To understand if the activities of GM-CSF are specific to persister cells, the total population (without persister isolation; more than 99% are normal cells) from exponential phase and stationary phase cultures of PAO1 and PD0300 were also treated with GM-CSF following the same procedure. Similar to persister cells, GM-CSF alone did not affect the viability of PAO1 and PDO300 normal cells (p > 0.1 for all conditions tested). However, unlike persister cells, GM-CSF did not exhibit any synergistic effects with antibiotics (ciprofloxacin and tobramycin tested) in killing normal cells of these two strains (p > 0.1 for all conditions tested; data not shown).

### GM-CSF is not effective against *E. coli*

**Figure 5:**
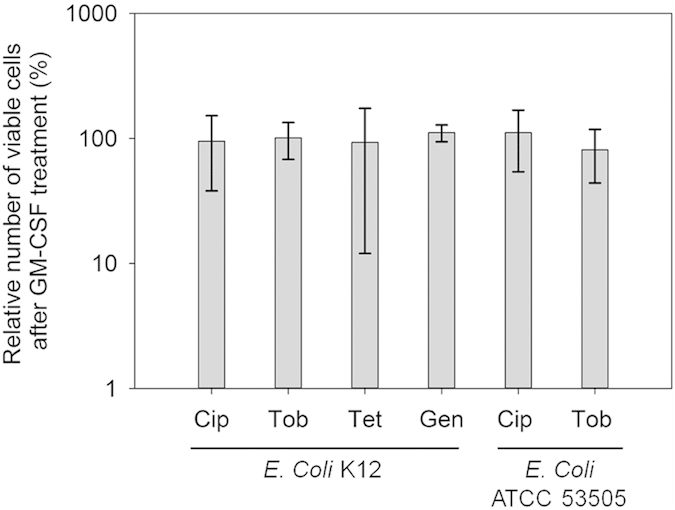
GM-CSF did not sensitize the persister cells of *E. coli* K12 and *E. coli* ATCC 53505 to antibiotics. The persister cells were isolated from exponential phase cultures of *E. coli* K12 and *E. coli* ATCC 53505 by killing the normal cells with 100 μg/mL ampicillin and 20 μg/mL ofloxacin (ATTCC 53505 is resistant to ampicillin, data not shown) respectively for 3.5 h, and then treated with or without 0.17 pM GM-CSF for 1 h, followed by adding an antibiotic as indicated and incubating for 3.5 h. The amount of BSA (contained in GM-CSF stocks) was adjusted to be the same in all samples. Following the treatment, the cell viability was determined by counting CFU. Cip: 2 μg/mL ciprofloxacin. Tob: 70 μg/mL tobramycin. Tet: 20 μg/mL tetracycline. Gen: 200 μg/mL gentamicin. The samples were tested in triplicate (n = 3). The number of CFU in the GM-CSF free sample (antibiotic only) varied depending on the antibiotic used, so the CFU of corresponding GM-CSF free control for each antibiotic was normalized as 100% for the convenience of comparison.

To understand if the observed activities of GM-CSF are species specific, we also tested it on *E. coli* strains including the lab strain *E. coli* K12^36^ and a pathogenic strain ATCC 53505^37^ associated with human urinary tract infection. The concentration of antibiotics which showed 2 log reduction in CFU of normal cells was selected for the experiments. Unlike *P. aeruginosa* PAO1 and PDO300, GM-CSF did not exhibit significant effect on the planktonic persister cells of *E. coli*. For example, treatment with 0.17 pM GM-CSF did not change the susceptibility of *E. coli* K12 persister cells to 2 μg/mL ciprofloxacin (p = 0.93) or 70 μg/mL tobramycin (p = 0.95) (Fig. 5). Similar results were found for the pathogenic *E. coli* ATCC 53505. As shown in Fig. 5, GM-CSF did not sensitize the persister cells isolated from exponential phase cultures to 2 μg/mL ciprofloxacin (p = 0.55) or 70 μg/mL tobramycin (p = 0.31). These results indicate that GM-CSF is not effective on the persister cells of *E. coli* K12 and ATCC 53505.

### Effects of GM-CSF on gene expression in *P. aeruginosa* PAO1

**Figure 6:**
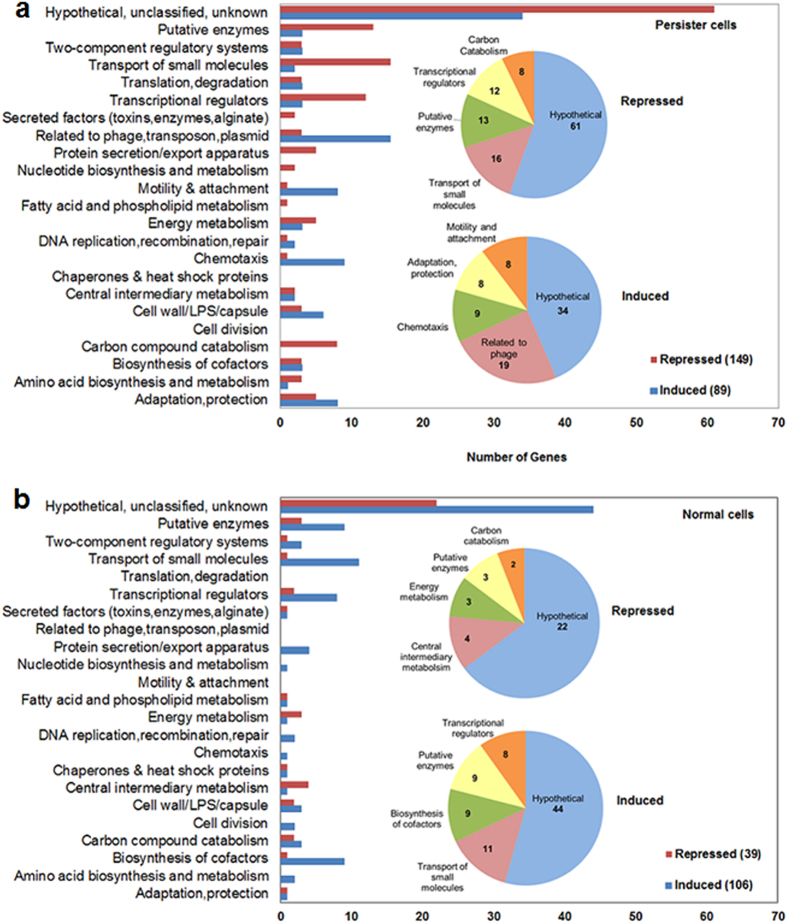
Effect of 0.17 pM GM-CSF on gene expression in *P. aeruginosa* PAO1. (**a**) Number and categories of genes that were consistently induced or repressed in two biological replicates of *P. aeruginosa* PAO1 persister cells. (**b**) Number and categories of genes that were consistently induced or repressed in two biological replicates of *P. aeruginosa* PAO1 normal cells.

To better understand GM-CSF induced sensitization of persisters to antibiotics, DNA microarrays were used to compare gene expression profiles of *P. aeruginosa* PAO1 persister cells with and without 1 h treatment with 0.17 pM GM-CSF. The results show that a total of 89 genes were induced and 149 genes were repressed by GM-CSF more than 1.5-fold (linear ratio) in both biological replicates (Fig. 6a). The induced genes include 34 genes coding for hypothetical proteins, 19 bacteriophage-like (pyocin) genes, 10 chemotaxis genes, 8 motility genes, and 18 genes with other functions (Fig. 6a). The repressed genes include 61 genes coding for hypothetical proteins, 16 genes related to the transport of small molecules, and 12 genes encoding transcriptional regulators (Fig. 6a). Table 2 shows the expression fold change of some representative genes based on the DNA microarray results of two biological replicates.

The gene expression patterns showed that a group of genes involved in motility and flagella synthesis were induced, including *flgBCDEFGHIJK, fliACDGMN*, and *cheYZ*. In addition, GM-CSF at 0.17 pM level was found to induce a large number of pyocin genes. For example, the pyocin regulatory gene *prtN* was induced by 2.8-fold compared to the GM-CSF free control. A large number of R-pyocin (PA0617–22, PA0625–30) and F-pyocin (PA0631–35, PA0637–38, PA640) related genes were also induced. The R and F pyocins are bacteriocins produced by *P. aeruginosa*^38^. Both the R and F-type pyocins can cause cytoplasmic membrane depolarization of sensitive strains by pore formation^38^. The R-type pyocins are comprised of an outer sheath, inner core, a baseplate, and 6 tail fibers. While the F-pyocins are devoid of an outer sheath, they do have a core, baseplate and tail fibers with short or long filaments^38^,^39^,^40^. Pyocin production can be provoked by DNA damage, and it is believed that the pyocinogenic bacteria can gain predominance by producing pyocins to eliminate pyocin-sensitive species in a mixed bacterial population^41^.

There were also a large number of genes repressed in response to GM-CSF. For example, *wbpK* (encoding for NAD-dependent epimerase/dehydratase), *algAL* (involved in alginate production), *phnA* (encoding for anthranilate synthase), and *str* (encoding for streptomycin 3 -phosphotransferase) were repressed by GM-CSF, suggesting that cell wall mediated protection and antibiotic resistance may be repressed.

qPCR was used to validate the DNA microarray results including 10 representative genes. Consistent results were obtained for 9 of these 10 genes, except for *recA* which was induced by 2.2-fold in DNA microarray data but was not significantly changed according to qPCR results (Supplementary Table S2). To understand if the effects of GM-CSF on *P. aeruginosa* PAO1 are specific to persister cells, DNA microarrays were also used to study the effects of 0.17 pM GM-CSF on the total population (>99% are normal cells) of *P. aeruginosa* PAO1 since GM-CSF only sensitized the persister subpopulation to antibiotics. Using the same 1.5-fold change as the cut off ratio, 106 genes were found induced and 39 genes were found repressed consistently in both biological replicates. The induced genes include 44 genes coding for hypothetical proteins, 11 genes associated with the transport of small molecules, 9 genes related to the biosynthesis of cofactors, 9 genes encoding putative enzymes, and 8 genes encoding transcriptional regulators (Fig. 6b). Unlike the results of persister cells, however, there was no induction of motility and phage related genes in normal cells. Among the repressed genes, there were 22 genes coding for hypothetical proteins, 4 genes related to metabolism, 3 genes associated with energy metabolism, and 3 genes encoding putative enzymes (Fig. 6b). The microarray data of normal cells were also validated by qPCR involving 4 representative genes: *yrfI* (induced)*, dnaB* (induced), PA0364 (repressed), and PA5548 (repressed) (Supplementary Table S3). These results indicate that GM-CSF has very different effects on persister cells and normal cells of *P. aeruginosa* PAO1, consistent with the CFU data.

### Supernatants from *P. aeruginosa* PAO1 persister cells treated with GM-CSF induced the killing of R2-pyocin sensitive *P. aeruginosa* PAK

**Figure 7:**
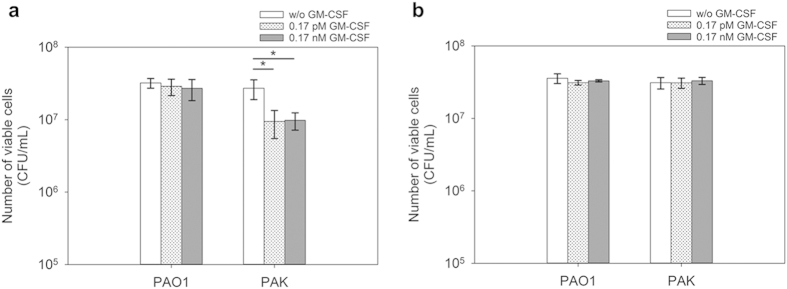
Effects of supernatants of GM-CSF treated *P. aeruginosa* PAO1 persister cell suspensions. The normal cells harvested from stationary phase cultures of *P. aeruginosa* PAO1 and PAK were treated with the supernatants collected from the persister cell suspensions of *P. aeruginosa* PAO1 (**a**), and PA0620::*phoA* (**b**) after treatment with 0.17 pM or 0.17 nM GM-CSF for 2 h. The amount of BSA (0.1%) was adjusted to be the same for all samples during GM-CSF treatment. Following the treatment, the viability of PAO1 and PAK cells was determined by counting CFU. The samples were tested in triplicate (n = 3). Error bars represent SD; *p < 0.05, one-way ANOVA followed by Tukey test.

R-type pyocins produced by *P. aeruginosa* strains can be categorized into five types termed R1 to R5^42^. Kohler *et al*.^39^ showed that the R1-pyocin producing *P. aeruginosa* PAK strain is susceptible to the R2-pyocins produced by *P. aeruginosa* PAO1. We found that treatment with GM-CSF induced R-pyocin related genes in *P. aeruginosa* PAO1 persister cells including PA0617, PA0619–22, and PA0625–30. Further test showed that, after *P. aeruginosa* PAO1 persister cells were treated with GM-CSF at 0.17 pM or 0.17 nM, the supernatants from the treated cell samples killed 65.3 ± 14.5% (p = 0.0201) and 67.8 ± 9.7% (p = 0.0132) of normal cells of *P. aeruginosa* PAK, respectively, compared to the GM-CSF free control (Fig. 7a). In contrast, no significant difference (p > 0.1) was observed for the same treatment of the normal cells of R2-pyocin resistant *P. aeruginosa* PAO1 (Fig. 7a). Moreover, when the persister cells of PA0620::*phoA*, an isogenic deletion mutant of PA0620 encoding the R2-pyocin tail fiber protein, were treated with 0.17 pM or 0.17 nM GM-CSF, the supernatants did not change the viability of *P. aeruginosa* PAO1 and PAK (Fig. 7b). Collectively, these results indicate that pyocin production in PAO1 might have been induced by GM-CSF. To our best knowledge, the interaction between *P. aeruginosa* and GM-CSF in pyocin production has not been explored to date.

## Discussion

It is well documented that GM-CSF is a vital cytokine for the host to fight invading pathogens^22^,^23^,^43^. However, little is known about the direct effects of GM-CSF on bacteria in the absence of host immune cells and the effects on persister cells have not been investigated. Persister formation is important to the survival of bacteria in competitive environments. Interestingly, the data from our study suggest that the human cytokine GM-CSF is effective in sensitizing the persister cells of *P. aeruginosa* to multiple antibiotics (especially ciprofloxacin and tobramycin), while these antibiotics alone are ineffective against persister cells. Such synergy and the microarray results suggest that there are previously unknown interactions between bacterial persister cells and GM-CSF besides the recruitment and activation of leukocytes by this host immune factor. The observed killing effects are less than 2 log and less than 1 log for most cases. However, it is worth noting that the effects are on persister cells, which are highly tolerant to essentially all conventional antibiotics. Further study of the underlying mechanism may reveal new strategies for persister control and help develop better therapeutic agents to eliminate persister cells.

Persister cells achieve dormancy by overproduction of proteins or toxins that inhibit the normal cellular processes^2^. As shown in this study, GM-CSF alone does not have significant effect on the viability of *P. aeruginosa* persister cells; however, 1 h treatment of *P. aeruginosa* PAO1 persister cells with 0.17 pM GM-CSF up-regulated 18 genes associated with motility and chemotaxis consistently in both biological replicates of the microarray experiments, including *flgBCDEFGHIJK* that are associated with the flagellar structure (flagellar basal body proteins, hook proteins and rod proteins)^44^,^45^. The induced genes *fliACDGMN* and *cheYZ* are associated with flagellar structure, motor switch, and clockwise/counterclockwise direction control of the flagellar rotation^46^,^47^,^48^,^49^. The induction of flagella/motility genes by GM-CSF suggests that *P. aeruginosa* persister cells can possibly respond to this host cytokine to move away from it, which can increase bacterial activity and thus lead to higher susceptibility to antibiotics. It will be interesting to test the effects of GM-CSF on the membrane potential of persister cells and normal cells.

The up-regulation of R and F-pyocin genes suggest that GM-CSF treatment may induce the production of bacteriocins, which are usually inducible by mutagenic agents like mitomycin C^38^. In total, 19 pyocin genes were induced involving both R-type and F-type pyocin genes. The lipopolysaccharides found in the outer membrane of *P. aeruginosa* cells act as receptors of R-type pyocins^38^,^39^,^50^. The killing mechanism of R-type pyocins involves membrane depolarization by pore formation^38^,^39^,^50^. The R2-pyocin genes found induced by GM-CSF are involved in the formation of base plate (PA0617-18), tail fiber (PA0619–21), tail sheath (PA0622), and tail (PA0625–28)^51^. The R-type pyocins have a higher bactericidal activity than F-type pyocins^38^. The induced F2-pyocin genes have functions in the formation of tail (PA0635) and baseplate (PA0637, PA0638, PA0640)^52^. Moreover, the gene *prtN* showed an up-regulation by 2.8-fold in two DNA microarray runs, which was confirmed by qPCR analysis (3.3-fold induction). This gene encodes an activator of pyocin genes^53^.

We speculate that GM-CSF might be creating stress on the persister cells of *P. aeruginosa* and inducing pyocin-related genes. R-type pyocins have the ability to rapidly kill target cells by binding to the bacterial cell surface through their tail fiber, followed by contraction of the pyocin sheath and penetration of the core through bacterial membranes^38^,^41^. The tail fiber protein is required for binding to the receptors of sensitive cells and contraction of tail is necessary for bacterial killing^54^,^55^. *P. aeruginosa* PAO1 produces R2-pyocins that kill R2-pyocin sensitive *P. aeruginosa* strains^39^,^51^; while the R2-pyocin producing strains are resistant to the R2-pyocins^51^. Here we observed that when the persister cells of *P. aeruginosa* PAO1 were treated with 0.17 pM or 0.17 pM GM-CSF for 2 h, the supernatants collected after centrifugation exhibited cidal effects on the normal cells of R2-pyocin sensitive strain *P. aeruginosa* PAK. No such effect was observed on the normal cells of R2-pyocin producing *P. aeruginosa* PAO1. These results support the DNA microarray data which show that GM-CSF induces R2-pyocin related genes (PA0617, PA0619–22, PA0625–30)^51^, and suggest that GM-CSF treatment may induce R2-pyocin production in PAO1 persisters under our experimental conditions. This can also affect the physiological stage of persister cells and thus enhance the killing by antibiotics.

In addition to the induced genes, qPCR also confirmed the repression of *wbpK*, an NAD-dependent epimerase/dehydratase gene involved in cell envelope biogenesis and catabolism, and *algA*, an alginate biosynthesis gene (mannose-1-phosphate guanylyltransferase/mannose-6-phosphate isomerase) which produces a precursor for alginate polymerization^56^. Moreover, Belanger *et al*.^57^ indicated that *wbpK* plays a role in O-antigen biosynthesis. The O-antigens are immunogenic, eliciting a strong antibody response from the infected host^57^. On the bacterial side, O-antigens have been shown to protect the bacteria from phagocytosis during *P. aeruginosa* infections^58^. Repression of *algA* may lead to lowered alginate layer formation, which in turn could reduce protection against host immune defenses and antibiotics^59^. The repression of *algL* (involved in degradation of mislocalized alginate) indicates possible abnormality in the alginate production, which may also help explain the enhanced killing by antibiotics^60^. In cystic fibrosis patients with *P. aeruginosa* infections, pyocyanin (encoded by *phnA* and *phnB* genes) is secreted by this bacterium leading to generation of superoxide and H_2_O_2_ in the infected lungs^61^,^62^. The repression of *phnA* gene by GM-CSF treatment suggests possibly lowered pyocyanin production, which may reduce virulence.

Compared to the activities in sensitizing planktonic persister cells, biofilm matrix degrading enzymes (DNase I and alginate lyase) were required for activities on biofilms of *P. aeruginosa* PAO1 and PDO300, respectively. In cystic fibrosis patients, during prolonged infection and exposure to antibiotics, some non-mucoid strains of *P. aeruginosa* mutate to mucoid strains, causing alginate overproduction^27^. The reduced effect of GM-CSF by alginate is consistent with this observation and provides new insights in the pathogenesis of *P. aeruginosa* infections. Our results suggest that developing new delivery strategies to allow GM-CSF to penetrate the extracellular matrix of biofilms might increase the efficacy of antibiotic therapies.

The mechanism of this new phenomenon deserves further investigation, which is part of our ongoing efforts. Nevertheless, the results of this study suggest that the host immune factors may directly interact with bacterial persister cells, leading to increased susceptibility to antibiotics. Alternatively, this may indicate that certain bacterial species can sense host immune factors and adjust physiology and virulence, as seen in the induction of chemotaxis and pyocin genes in *P. aeruginosa* PAO1 persister cells by GM-CSF and the lack of activities against *E. coli* persister cells. These data suggest that *P. aeruginosa* may be able to sense GM-CSF and leave the persister stage (induction of chemotaxis genes, etc,). In the absence of antibiotics (e.g. without antibiotic therapy), such response would be beneficial for bacterial cells to avoid the attack by the host immune system and establish a successful infection. However, wakeup from persister stage will increase bacterial susceptibility to antibiotics, which are more effective against active cells. More effective antimicrobial chemotherapies (through synergies between immune factors/synthetic factors and antibiotics) may be developed by taking advantage of such activities to eliminate persister cells. *P. aeruginosa* has remarkable adaptability to different environmental niches and varying hosts^63^. Thus, further study of the interaction between *P. aeruginosa* cells and GM-CSF, and characterization of the effects of GM-CSF on other bacterial species will help understand how bacteria establish chronic infections and pave the path for developing more effective treatments.

## Materials and Methods

### Bacterial strains and growth media

The bacterial strains used in this study include two *P. aeruginosa* PAO1 strains (obtained from Prof. Thomas. K Wood^32^ and Prof. Matthew Parsek^33^, respectively), an isogenic mutant PDO300 (*mucA22)*, *E. coli* K12, and *E. coli* ATCC 53505^37^. The transposon mutant PA0620::*phoA* (with the transposon insertion IS*phoA*/hah) was obtained from the *P. aeruginosa* PAO1 mutant library at University of Washington^64^. Overnight cultures of these strains were prepared in Luria Bertani (LB) medium^65^ containing 10 g/L tryptone, 5 g/L yeast extract, and 10 g/L NaCl at 37 °C with shaking at 200 rpm. The cultures of PA0620::*phoA* were grown in LB medium supplemented with 60 μg/mL tetracycline. Recombinant human GM-CSF was purchased from R&D systems (Minneapolis, MN, USA). The stocks used in this study contained 10 μg/mL GM-CSF, dissolved in phosphate buffer saline (PBS) pH 7.4, supplemented with 0.1% bovine serum albumin (BSA).

### Kinetics of bacterial killing during antibiotic treatment

The antibiotic conditions used for isolation of persister cells of *P. aeruginosa* PAO1, PDO300, *E. coli* K12, and *E. coli* ATCC 53505 were validated by measuring the killing curves over time similar to the method described previously^66^,^67^. The exponential and/or stationary phase cultures were treated with 200 μg/mL ciprofloxacin (*P. aeruginosa* PAO1 and PDO300), 100 μg/mL ampicillin (*E. coli* K12) or 20 μg/mL ofloxacin (*E. coli* ATCC 53505) for 4.5 h, and the viability of cells was determined every 0.5 h during treatment by plating the cells on LB agar plates and counting CFU after 24 h of incubation at 37 °C using the drop plate method as described previously^68^. The stationary phase cultures were overnight cultures after 16 h of incubation, while the exponential cultures were harvested from subcultures when optical density at 600 nm (OD_600_) reached 0.3 to 0.4. To identify the minimum inhibitory concentration (MIC) of selected antibiotics, the overnight cultures of *P. aeruginosa* PAO1, PDO300, and *E. coli* K12 were subcultured with an initial OD_600_ of 0.1 in LB medium in 96-well plates. Each antibiotic (ciprofloxacin, tobramycin, tetracycline, and gentamicin) was tested at different concentrations (from 0.1 to 204.8 μg/mL) in triplicate. The OD_600_ was recorded at 0, 1, 2, 3, 4, 5, 6, 7, and 18 h after inoculation using a plate reader (BioTek Instruments Inc., Winooski, VT). MIC was determined as the lowest concentration at which no growth of a bacterial strain was observed after overnight incubation.

### Effects of GM-CSF on planktonic cells

The experiments performed on the planktonic cells were conducted with cells harvested from both exponential and stationary phase (16 h after inoculation) cultures. For stationary cultures, the cells from an overnight culture of *P. aeruginosa* PAO1 in LB medium were collected by centrifuging at 8,000 rpm for 10 min and washed twice with PBS (pH 7.4). The washed cells were resuspended in 25 mL PBS buffer and vortexed gently for 1 min to separate cells. A portion of the sample was used to determine the viability by plating the cells on LB agar plates and counting CFU using the drop plate method, as described previously^68^, while the rest was used for isolation of persisters by adding 200 μg/mL ciprofloxacin for *P. aeruginosa* PAO1 and PDO300, 100 μg/mL ampicillin for *E. coli* K12, and 20 μg/mL ofloxacin for *E. coli* ATCC 53505, and incubating at 37 °C for 3.5 h with shaking at 200 rpm^8^,^31^. After incubation, the antibiotic was washed away with PBS buffer by centrifuging thrice at 4 °C, 8,000 rpm for 10 min each, and vortexed for 1 min after adding PBS. To test the effects of GM-CSF on viability of *P. aeruginosa*, the washed cells were transferred to microcentrifuge tubes, with 1 mL of washed cells in each tube. GM-CSF was added at 0, 0.17, 1.7, and 17 pM to treat the cells for 1 h. These concentrations were selected because 0.17 pM is the concentration of GM-CSF found in the blood plasma of healthy human beings^19^. The amount of BSA (0.1%) was adjusted to be the same for all samples so that the effects of GM-CSF can be studied specifically. After the one hour treatment, the viability of persister cells was determined by counting CFU. To study the synergy with antibiotics, the GM-CSF with or without an antibiotic was added to the persister cells and incubated for 3.5 h. Ciprofloxacin and tobramycin (both at 5 μg/mL) were tested. Thereafter, the cells were washed by centrifuging thrice at 13,200 rpm for 2 min and resuspending in 1 mL PBS (pH 7.4). The viability of the cells was determined by counting CFU using the drop plate method.

For exponential phase planktonic cells, the experiments were performed with cells harvested from exponential phase subcultures with an OD_600_ of 0.3 to 0.4. Briefly, after preparing an overnight culture of the tested strain in 25 ml LB medium, a subculture in LB medium was inoculated with an OD_600_ of 0.01. The subculture was incubated at 37 °C with shaking at 200 rpm for 3–4 h, till OD_600_ reached 0.3 to 0.4. After washing the subculture twice by centrifuging at 8,000 rpm for 10 min with PBS (pH 7.4) and isolating persister cells as described above, sequential treatment was performed for 1 h with or without 0.17 pM GM-CSF, followed by addition of an antibiotic and further incubation for 3.5 h. Cell viability was determined after treatments using drop plate method as described above. The antibiotics (ciprofloxacin, tobramycin, tetracycline, and gentamicin) used for exponential phase cultures were added at a concentration of 200 μg/mL for both *P. aeruginosa* PAO1 and PDO300. To confirm that any change in the viability of bacterial cells is due to the specific effect of GM-CSF rather than any contaminant, the persister cells of *P. aeruginosa* PAO1 isolated from exponential phase cultures were treated with 0.17 pM GM-CSF in the presence of different concentrations of anti-GM-CSF antibody (0, 17, and 170 pM). The persister cells underwent treatment with GM-CSF alone, anti-GM-CSF alone, or GM-CSF neutralized by anti-GM-CSF for 2 h. Five μg/mL ciprofloxacin was then added to all samples, which were incubated for another 3.5 h. After washing the cells thrice at 13,200 rpm for 2 min, CFU was counted using drop plate method.

The effect of GM-CSF was also compared with heat-inactivated GM-CSF by treating the persister cells of *P. aeruginosa* PAO1 isolated from a stationary phase culture with 0.17 pM GM-CSF and heat-inactivated 0.17 pM GM-CSF, in the presence of 5 μg/mL ciprofloxacin with incubation for 3.5 h. The heat-inactivation of GM-CSF was carried out by heating GM-CSF overnight at 75 °C. After treatment of *P. aeruginosa* PAO1 persister cells with GM-CSF and ciprofloxacin, the cells were washed thrice with centrifugation at 13,200 rpm for 2 min, followed by CFU count using drop plate method.

### Effect of GM-CSF on biofilm cells

After preparing an overnight culture, each bacterial strain tested for biofilm formation was subcultured to an initial OD_600_ of 0.01 in a petri dish containing 20 mL LB medium and sterile 316L stainless steel coupons (1.75 cm × 1 cm, 0.05 cm thick). The biofilms were grown for 24 h at 37 °C without shaking. After incubation, the coupons were washed by gently dipping in PBS and placed in 12-well plates. There were 7 treatment conditions in total and each condition was tested in triplicate: (i) GM-CSF alone, (ii) antibiotic alone, (iii) GM-CSF and alginate lyase, (iv) GM-CSF and antibiotic, (vi) antibiotic and alginate lyase, and (vii) GM-CSF, antibiotic, and alginate lyase. In all the experiments, the concentrations of GM-CSF and alginate lyase were kept at 0.17 pM and 50 μg/mL, respectively. The control samples were supplemented with the same amount of BSA (0.1%) as present in the samples with 0.17 pM GM-CSF. The coupons were incubated at 37 °C for 3.5 h. After treatment, each coupon was gently washed with PBS and placed in a test tube containing 3 mL of PBS. The coupons were gently sonicated (B200, Sinosonic Industrial Co., Ltd., Taiwan) for 4 min to release the biofilm cells from the coupon surface. This condition was confirmed to not kill the cells^69^. After vortexing for 1 min, the cell suspensions were plated on LB agar plates using drop plate method to count the number of CFU after incubation at 37 °C for 24 h.

### DNA microarray analysis

The RNA from persister cells was isolated by following our protocol as described previously^70^. The persister cells of *P. aeruginosa* PAO1 were isolated from 60 mL overnight cultures by adding 200 μg/mL ciprofloxacin and incubating at 37 °C for 3.5 h with shaking at 200 rpm. The isolated persister cells were washed with PBS and resuspended in 300 mL PBS. These persister cells were supplemented with 0.17 pM GM-CSF (treatment) or the same amount of BSA (0.1%), but no GM-CSF (control). The control and the treatment samples were incubated at 37 °C for 1 h with shaking at 200 rpm. After incubation, the cells were quickly collected by centrifugation at 10,000 rpm for 2 min at 2 °C. The supernatant was decanted and the cell pellets were flash frozen in a dry ice-ethanol bath. Then cell pellets were stored at −80 °C until RNA isolation. Total RNA was isolated using RNeasy Mini kit (Qiagen, Valencia, CA, USA) including on-column DNase treatment, and sent to the DNA microarray facility at SUNY Upstate Medical University (Syracuse, NY, USA) to check on a bioanalyzer before hybridization on GeneChip *P. aeruginosa* Genome Arrays (Affymetrix, Santa Clara, CA, USA). GeneChip Operating Software (MAS 5.0) was used to identify the differentially expressed genes by signal detection based on Wilcoxon signed rank test and Tukey’s biweight. The fold change for each gene was calculated as a ratio of treatment to control signals. In comparison, similar DNA microarray analysis was also performed on the total population from stationary cultures of *P. aeruginosa* PAO1. All RNA samples used in this study for DNA microarray and qPCR experiments had a RNA integrity number (RIN) > 9.0. RIN estimates the integrity of RNA and its value ranges from 10 (completely intact) to 1 (totally degraded)^71^. Contamination of normal cell mRNA in persister samples was not a concern, since ciprofloxacin is known to trigger cell autolysis in Gram-negative bacteria causing mRNA degradation of the dead cells^72^,^73^,^74^,^75^. Microarray data has been deposited in Gene Expression Omnibus (GEO: GSE63588), compliant with Minimum Information About a Microarray Experiment (MIAME) guidelines.

### Quantitative real-time PCR (qPCR) analysis

To validate the DNA microarray results, the transcriptional levels of 10 representative genes were also tested using qPCR, including seven induced genes (*flgF, prtN, fliN*, PA0620, PA0633, *recA* and PA0640), two repressed genes (*wbpK, algA*), and one unchanged gene (*argH*). The gene *rpoD* (RNA polymerase sigma factor RpoD) was selected as housekeeping gene for the qPCR study as described previously^76^. For the normal cells (no persister isolation), qPCR was performed on two induced genes (*yrfI*, *dnaB*) and two repressed genes (PA0364, PA5548) to confirm the microarray results. The cDNA was synthesized from the isolated RNA of control and treatment samples using iScript™ cDNA Synthesis Kit (Biorad, Hercules, CA, USA). The primers were designed using OligoPerfect™ Designer (Life Technologies, Grand Island, NY, USA) and Primer blast (NCBI) to obtain products with sizes between 231 and 350 bp, and melting temperatures between 59.5 and 60.3 °C. The sequence and the product size for each primer pair are listed in Supplementary Tables S4 and S5. The qPCR samples were prepared by mixing cDNA, primers, and iTaqTM Universal SYBR Green Supermix (Biorad, Hercules, CA, USA). The qPCR reactions were performed using an Eppendorf Mastercycler Realplex thermal cycler (Eppendorf, Hauppauge, NY, USA). The qPCR reactions underwent the following cycles: heat activation at 95 °C for 2 min, 40 cycles of denaturation at 95 °C for 15 s, and annealing/extension at 60 °C. A melting curve was added after the PCR cycle as a dissociation analysis to confirm if the PCR reaction produced only the desired product. The melting curve was set at 95 °C for 15 s, 50 °C for 30 s, 20 min hold with temperature gradient, and 95 °C for 15 s. After the qPCR reactions, the expression ratios of the selected genes were analyzed using LinReg PCR program (Heart Failure Research Center, Amsterdam, Netherlands).

### Effect of GM-CSF on the pyocin production in *P. aeruginosa* PAO1 persister cells

The planktonic cells were harvested from overnight cultures (16 h after inoculation) of *P. aeruginosa* PAO1 and PAK grown in 30 mL LB medium. After washing the cells with PBS (pH 7.4) by centrifuging at 8,000 rpm for 10 min twice at room temperature, the cells were resuspended in 30 mL PBS for testing the effects of supernatants from GM-CSF treated cell suspensions. To isolate persister cells of *P. aeruginosa* PAO1, the washed 15 mL overnight culture was treated with 200 μg/mL ciprofloxacin for 3.5 h at 37 °C, with shaking at 200 rpm. Thereafter, the ciprofloxacin was washed away from the isolated persister cells by centrifugation for three times at 8,000 rpm for 10 min at 4 °C and resuspended in 15 mL PBS.

Then, the washed cells were transferred to centrifuge tubes, with 5 mL of washed persister cells in each tube. GM-CSF was added at 0.17 pM or 0.17 nM. Samples were adjusted to make sure they all have the same amount of BSA (0.1%) as in the GM-CSF stock. The samples were then incubated at 37 °C for 2 h with shaking at 200 rpm. The supernatant for testing the presence of pyocin was collected by centrifugation at 13,200 rpm for 5 min at 4 °C. To ensure the supernatant does not contain any bacterial cells, it was filtered using a 0.2 μM nylon filter. The sterile supernatants were added at a volume of 100 μL to each microcentrifuge tube with a total volume of 1 mL washed normal cells (~10^7^ cells) of *P. aeruginosa* PAO1 or PAK, and the samples were incubated at 37 °C for 3.5 h with shaking at 200 rpm. After washing the cells three times with PBS, the viability was determined using the drop plate method. For comparison, a similar experiment was also performed with a mutant of R2-pyocin tail fiber (PA0620::*phoA*).

### Statistical Analyses

The CFU data were analyzed with one-way ANOVA followed by Tukey test (when needed) throughout the study using SAS 9.2 software (SAS Institute, Cary, NC, USA). The results with p < 0.05 were considered significant.

## Additional Information

**How to cite this article**: Choudhary, G. S. *et al*. Human Granulocyte Macrophage Colony-Stimulating Factor Enhances Antibiotic Susceptibility of *Pseudomonas aeruginosa* Persister Cells. *Sci. Rep*. **5**, 17315; doi: 10.1038/srep17315 (2015).

## Supplementary Material

Supplementary Information

## Figures and Tables

**Table 1:** Viability of *P. aeruginosa* PAO1 persister cells isolated from stationary cultures after treatment with GM-CSF and/or selected antibiotics.

Antibiotic	Change in viability (compared to antibiotic alone)			
	GM-CSF Concentration			
	0 pM	0.17 pM	1.7 pM	17 pM
No antibiotic	−	NC	NC	NC
Ciprofloxacin (5 μg/mL)	NC	−61.5 ± 14.5% ^**^	−53.8 ± 16.1%^**^	−74.0 ± 2.9%^***^
Tobramycin (5 μg/mL)	NC	−77.1 ± 2.0%^***^	−82.7 ± 2.3%^***^	−86.5 ± 1.7%^***^

**p < 0.01, ***p < 0.001, one-way ANOVA followed by Tukey test. NC: No significant change.

**Table 2:** Expression fold change of representative genes in *P. aeruginosa* PAO1 persister cells based on the average of two DNA microarray runs.

Gene	Expression fold change	Functions
*flgBFH*	+(2.1–2.6)	Motility & Attachment; Cell wall/LPS/capsule
*cheYZ*	+(2.1–2.2)	Chemotaxis
*fliN*	+2.3	Motility & Attachment; Chemotaxis; Adaptation, protection
*prtN*	+2.8	Pyocin regulatory gene
PA0618	+2.8	R-pyocin bacteriophage
PA0619	+2.8	R-pyocin bacteriophage
PA0620	+2.7	R-pyocin bacteriophage
PA0625	+2.8	R-pyocin bacteriophage
PA0633	+3.1	F-pyocin bacteriophage
PA0638	+2.9	F-pyocin bacteriophage
PA0640	+3.1	F-pyocin bacteriophage
*wbpK*	−5	NAD-dependent epimerase/dehydratase
*algL*	−2.5	poly(beta-D-mannuronate) lyase
*algA*	−2.5	phosphomannose isomerase/guanosine 5′-diphospho-D-mannose pyrophosphorylase
