# Supplementary material for: Human Granulocyte Macrophage Colony-Stimulating Factor Enhances Antibiotic Susceptibility of Pseudomonas aeruginosa Persister Cells
Source: Sci Rep. 2015 Nov 30;5:17315. doi: 10.1038/srep17315 (PMC4663479; doi:10.1038/srep17315)
Supplement: Supplementary Information [file srep17315-s1.pdf]

Supplementary Information

Human Granulocyte Macrophage Colony-Stimulating Factor Enhances Antibiotic Susceptibility of *Pseudomonas aeruginosa* Persister Cells

Geetika S. Choudhary[1, 2], Xiangyu Yao[1, 2], Jing Wang[1, 2], Bo Peng [1,2], Rebecca A. Bader[1, 2], and Dacheng Ren[1,2,3,4,*]

[1]Department of Biomedical and Chemical Engineering, Syracuse University, Syracuse, NY 13244, USA.
[2]Syracuse Biomaterials Institute, Syracuse University, Syracuse, NY 13244, USA.
[3]Department of Civil and Environmental Engineering, Syracuse University, Syracuse, NY 13244, USA.
[4]Department of Biology, Syracuse University, Syracuse, NY 13244, USA.

[*]Corresponding author. Email: dren@syr.edu

Supplementary Figures

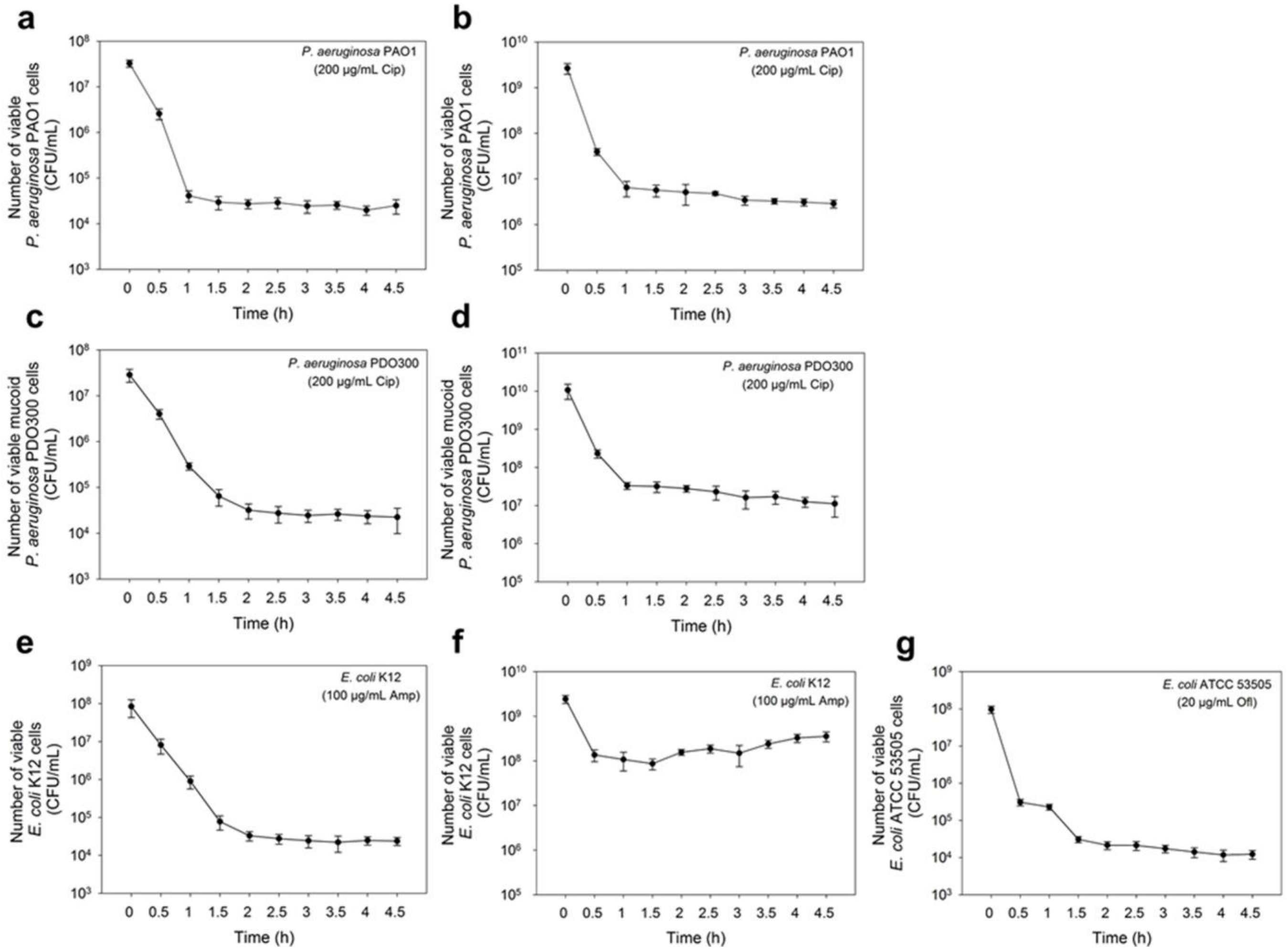

Supplementary Figure S1. Biphasic killing curves of *P. aeruginosa* PAO1, PDO300, *E. coli* K12, and *E. coli* ATCC53505 cells collected from exponential and stationary phase cultures. *P. aeruginosa* PAO1 cells collected from exponential (**a**) and stationary (**b**) phase cultures were treated with 200 µg/mL ciprofloxacin (Cip). *P. aeruginosa* PDO300 cells collected from exponential (**c**) and stationary (**d**) phase cultures were treated with 200 µg/mL ciprofloxacin (Cip). *E. coli* K12 cells collected from exponential (**e**) and stationary (**f**) phase cultures were treated with 100 µg/mL ampicillin (Amp). *E. coli* ATCC 53505 cells collected from exponential phase cultures (**g**) were treated with 20 µg/mL ofloxacin (Ofl). The samples were tested in triplicate (n=3). Error bars represent SD.

Supplementary Tables

Supplementary Table S1. MIC of ciprofloxacin, tobramycin, tetracycline, and gentamicin for strains *P. aeruginosa* PAO1, *P. aeruginosa* PDO300, and *E. coli* K12.

**Bacterial strain**	**Antibiotic MIC**			
	Ciprofloxacin	Tobramycin	Tetracycline	Gentamicin
P. aeruginosa PAO1	3.2 µg/mL	0.8 µg/mL	12.8 µg/mL	6.4 µg/mL
P. aeruginosa PDO300	6.4 µg/mL	3.2 µg/mL	25.6 µg/mL	6.4 µg/mL
E. coli K12	0.4 µg/mL	0.4 µg/mL	0.8 µg/mL	3.2 µg/mL

Supplementary Table S2. Comparison of qPCR results with the DNA microarray results for 10 representative genes in *P. aeruginosa* PAO1 persister cells.

**Gene**	**Expression fold change (GM-CSF vs. Control)**	
	DNA microarray	qPCR
flgF	+2.3	+3.3
fliN	+2.3	+8.5
prtN	+2.8	+3.3
PA0620	+2.7	+5.2
PA0633	+3.1	+6.5
PA0640	+3.1	+4.7
recA	+2.2	No significant change
wbpK	-5.0	-2.1
algA	-2.5	-5.9
argH	No significant change	No significant change

Supplementary Table S3. Comparison of qPCR results with the DNA microarray results for 4 representative genes in *P. aeruginosa* PAO1 normal cells.

**Gene**	**Expression fold change (GM-CSF vs. Control)**	
	DNA microarray	qPCR
dnaB	+8.2	+3.0
yrfI	+4.7	+4.3
PA5548	-5.5	-1.7
PA0364	-11.8	-35.5

Supplementary Table S4. Primers used for qPCR tests on PAO1 persister cells.

Primer	Sequence	Product size (bp)
rpoD Forward	CGGTCAACCTGAAGGACGAT	253
rpoD Reverse	ACCAGCTTGATCGGCATGAA	
flgF Forward	TCAACCCGAACCTGAAGCAG	273
flgF Reverse	CACCACGCTCAAGTGATTAGC	
fliN Forward	GGATGTGATCCTGGACATCCC	245
fliN Reverse	GCAGCTTCTTGATGCGTTCG	
prtN Forward	ACCGTGGAATTGGTCTACCG	271
prtN Reverse	CTCAGGATGCGATGCTGTCA	
PA0620 Forward	TGCTGTCTCGAACAGTAGCG	268
PA0620 Reverse	AGGAACCTCCAATGTCGCAG	
PA0633 Forward	CAGATCTACGCCCTGGTTCC	253
PA0633 Reverse	TCGTCTCGCCATCTTTCTCG	
PA0640 Forward	CGACATATTCAAGCGAGCCG	240
PA0640 Reverse	AGGTCAGCCCTTTCGATTCC	
recA Forward	TCGGAACATTCTTCCCGTCG	258
recA Reverse	CGATGTCCAGACCCAGGGAG	
wbpK Forward	ACAGGTCGATGCTTCCAAGG	243
wbpK Reverse	CCCCAGGTAGCGACAAATGA	
algA Forward	GTCGTCGATCTGGGACGTG	263
algA Reverse	GGTAGACCTCGCAGTGGTTC	
argH Forward	CAGGAAGACAAGGAACCGCT	231
argH Reverse	GCTGTCTACGCCGTACTTCA	

Supplementary Table S5. Primers used for qPCR tests on PAO1 normal cells.

Primer	Sequence	Product size (bp)
dnaB Forward	CGACGTGGAAACCACTCTGA	347
dnaB Reverse	CGATGGTCATGGCGGTAGAA	
yrfI Forward	CCCGCATGTCCCATTCAGAT	303
yrfI Reverse	CGATGGACTCCGCCGAATAG	
PA5548 Forward	TATTCTTCGTCGGCGTGACC	300
PA5548 Reverse	TGGTCGGGATGATGATTGCC	
PA0364 Forward	AGCTCTCGGTCTACGGTCTT	303
PA0364 Reverse	GAAAAGCCGTGAAGCACGTT	
